# Multisystem Thoracic Hydatidosis With Intracardiac Involvement in an Elderly Male Patient: A Case Report and Literature Review

**DOI:** 10.7759/cureus.88556

**Published:** 2025-07-22

**Authors:** Muhammad Ayub, Sobia Ahmed, Quang Dai La, Aiman Baloch, Noreen Ismail, Abdul Khaliq, Pari Gul, Francis Pryor

**Affiliations:** 1 Radiology, Bolan Medical Complex Hospital, Quetta, PAK; 2 Medicine, The Innovative STEMagazine, College Station, USA; 3 Biology, Texas A&M University, College Station, USA; 4 Medicine, Mekran Medical College Turbat, Balochistan, PAK; 5 Radiology, Bolan Medical College, Quetta, PAK; 6 Medicine, Lake Erie College of Osteopathic Medicine, Erie, USA

**Keywords:** cardiac, echinococcosis, echinococcus granulosus, hydatid disease, liver, parasitic infection, thoracic, transthoracic echocardiography

## Abstract

*Echinococcus granulosus* hydatid disease is an infestation by a parasite, commonly of the liver and lungs. Multicomponent and cardiac thoracic involvement are rare and diagnostic challenges. A report of a shortness-of-breath-impacted, chest-tightness-impacted man aged 80 years who presented with grape-like vesicles expectorated is described. The serological assay was echinococcosis-positive. Transthoracic echocardiography and contrast-enhanced CT revealed disseminated thoracic hydatid disease in the lungs, pleura, mediastinum, pericardium, and right ventricle. The patient was referred for cardiothoracic surgery. The case highlights the importance of extensive imaging in the diagnosis of rare presentations of hydatid disease and the need for increased clinical suspicion in endemic areas. Monitoring hydatid disease early in patients with cystic expectoration and clear chronologia is important, especially in endemic areas, and should be followed promptly with serological tests and imaging. In this case, echocardiography was useful in demonstrating intracardiac involvement, while CT imaging was able to establish the degree of thoracic dissemination. Both modalities were essential in assessing the extent of disease dissemination and the potential surgical plan. Clinicians should have hydatid disease on their differential diagnosis in patients with cystic expectoration without socratic/normal history and risk factors, as early evaluation is important in how they may care for the patient.

## Introduction

Cystic echinococcosis (CE), or hydatid disease, is a zoonotic parasitic infection caused by the larval form of *Echinococcus granulosus* [1]. It remains a significant public health problem in endemic regions, particularly where livestock farming is prevalent and veterinary control is deficient [2-3].

The most commonly affected organs are the lungs and liver, of which 70% of the cases involve the liver [4]. Involvement of the heart is less frequent in about 0.5% to 2% of all CE cases [5].

Intrathoracic echinococcosis involving multiple thoracic organs-such as lungs, pleura, mediastinum, and heart-is extremely rare [6]. Its multi-local nature renders it difficult to treat and diagnose due to its multiforme clinical manifestations and serious complications such as rupture of the cyst, anaphylaxis, and cardiac tamponade [7].

Intracardiac hydatid cysts may be asymptomatic for several years or present with nonspecific cardiopulmonary symptoms, and hence, early detection is not feasible in the absence of high suspicion and high-technology imaging [8].

This case report documents an unusual case of disseminated thoracic hydatidosis in an elderly male patient. Echinococcal cysts are observed in the lungs, pleura, mediastinum, pericardium, and right ventricle of the heart. This presentation emphasizes the importance of complete imaging and serological assessment for making complicated hydatid disease diagnoses, especially in endemic areas.

This paper was also previously presented as a poster on 13th April 2025 at the Joint Conference of the Body Imaging Radiological Society of Pakistan (BIRSP) and the National Breast Radiological Society of Pakistan (NBRSP).

## Case presentation

An 80-year-old male patient presented to the pulmonology outpatient clinic with deteriorating shortness of breath, chest tightness, and productive cough. Sputum was grape-like vesicular structures and translucent membranous fragments. There was no history of recent travel, livestock exposure, or past diagnosis of parasitic disease. The patient had no relevant comorbid disease and did not have past thoracic trauma or surgery. 

**Figure 1:**
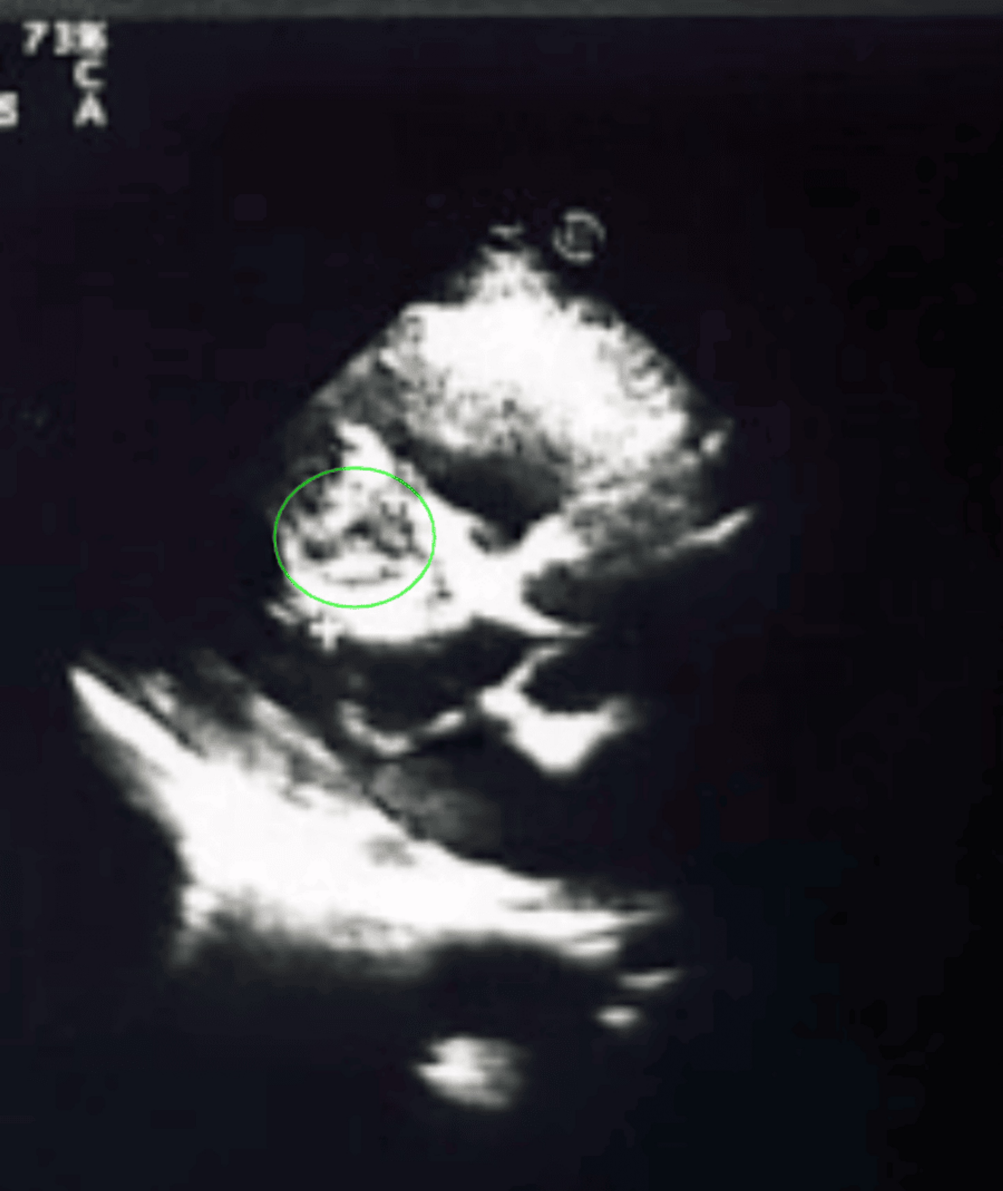
Transthoracic echocardiogram showing a cystic mass at basal interventricular septum.

Breath sounds were reduced bilaterally on auscultation, with diffuse wheezing. Laboratory results revealed positive serology for* Echinococcus granulosus. *Transthoracic echocardiography revealed a discrete cystic mass in the basal interventricular septum (Figure 1).

**Figure 2:**
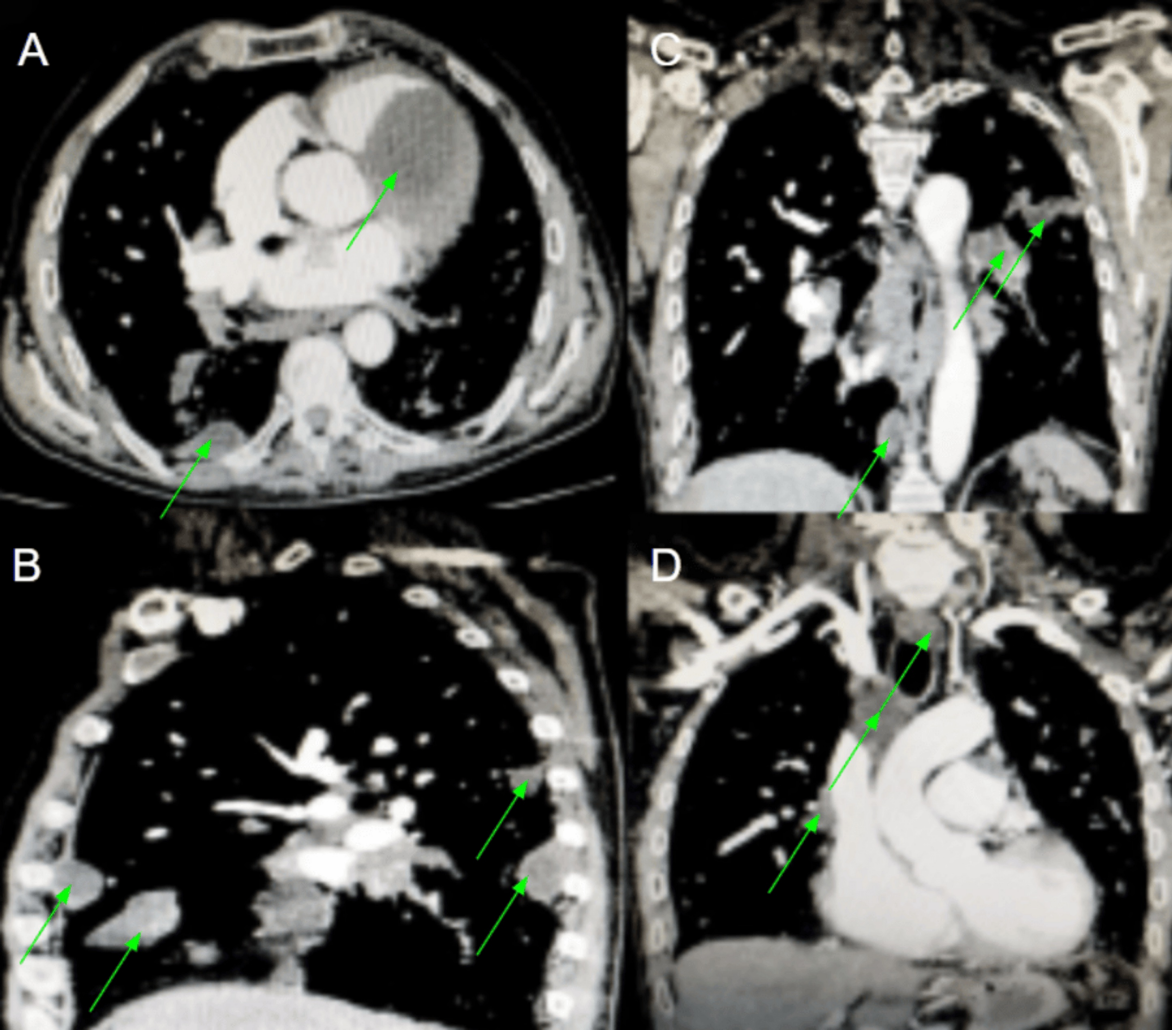
CECT images showing widespread mediastinal and pleuropulmonary hydatidosis including intracardiac, pericardial, and paravertebral hydatid cysts. Green arrow points to area of cysts. A. CECT chest axial image. B. CECT chest sagital reformatted image. C-D. CECT chest coronal reformatted images. CECT: Contrast-enhanced computed tomography

Further imaging with contrast-enhanced computed tomography (CECT) chest revealed extensive thoracic hydatid disease. Multiple cystic lesions were noted in both lung fields, pleural spaces, mediastinum, and pericardium. Surprisingly, a large cyst was noted within the right ventricle with firm attachment to the interventricular septum. There were some paravertebral hydatid cysts (Figure 2).

Because of the extensive intrathoracic and intracardiac disease, the patient was referred to the cardiothoracic surgical team for definitive care.

## Discussion

**Figure 3:**
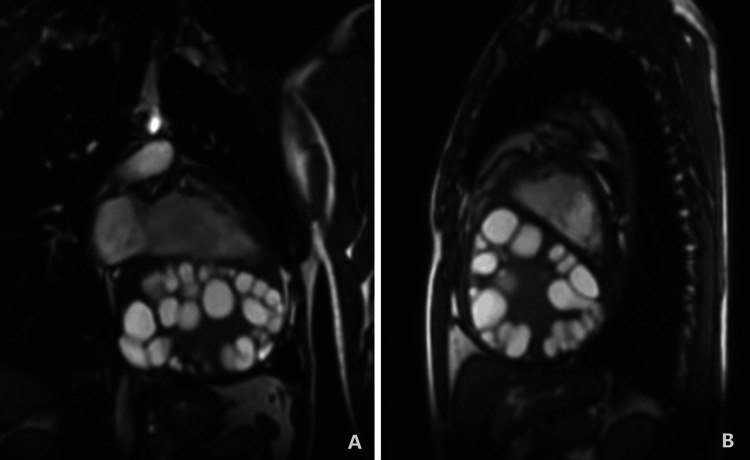
Cardiac magnetic resonance imaging through static images of a cine loop. (A) The sagittal section features a four-chamber view, while (B) displays a short-axis view. Both images depict multiple hypointense ring-shaped thick-walled hydatid cysts occupying the entire cavity of the left atrium and left ventricle. Image and caption reproduced from Lalani et al., 2024 [9] under the terms of the Creative Commons Attribution–NonCommercial 4.0 International License (https://creativecommons.org/licenses/by-nc/4.0/).

CE is caused by the larval stage of *Echinococcus granulosus*, and although liver and lung involvement are common, cardiac localization remains rare, with intracardiac cysts occurring in just 0.5-2% of patients [5]. Within the heart, the distribution of hydatid cysts varies, with the left ventricle being most commonly affected (55-60%) (Figure 3), followed by the right ventricle (10-15%), pericardium (7%), pulmonary artery (6-7%), left atrium (5%), right atrium (3-4%), and interventricular septum (~4%) [9-11]. Cardiac hydatidosis most frequently affects the left ventricle, possibly due to its rich coronary blood flow; right ventricular involvement, as in the current case, is uncommon and carries a higher risk of cyst rupture into the pulmonary circulation or pericardial space [12].

Intrathoracic extension to more than one compartment-lungs, pleura, mediastinum, and pericardium-is exceedingly rare and implies either primary hematogenous dissemination or secondary transdiaphragmatic spread from hepatic cysts [13]. Vesicular, grape-like congeries in sputum, or hydatidoptysis, are a pathognomonic but infrequent sign, most often the result of rupture of a pulmonary cyst into the bronchial tree [14].

**Figure 4:**
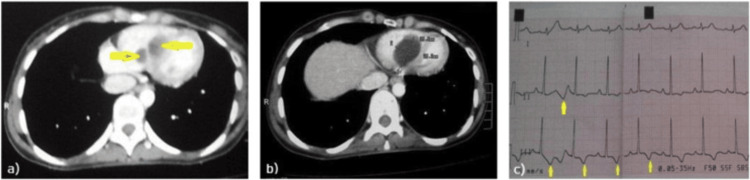
Contrast-enhanced computed tomography images showing (a) two well-defined filling defects (arrows) in the right ventricle with no enhancement after contrast administration and, (b) dimensions of the larger filling defect (cyst). (c) Electrocardiogram (ECG) showing T-wave inversion in the inferior leads (arrows) indicating ischaemic changes. Image and caption reproduced from Aljaber et al., 2020 [15] under the terms of the Attribution-NonCommercial-No Derivatives 4.0 International License (https://creativecommons.org/licenses/by-nc-nd/4.0/).

**Figure 5:**
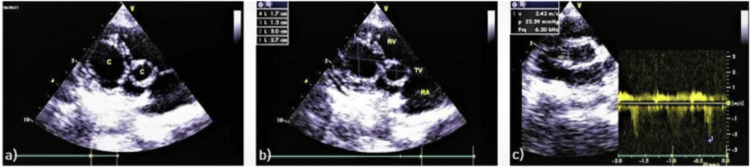
Transthoracic echocardiography images showing (a) two cystic lesions in the right ventricle, (b) dimensions of the two cysts and, (c) mild preoperative tricuspid regurgitation (right ventricular pressure 23 mmHg). Image and caption reproduced from Aljaber et al., 2020 [15] under the terms of the Attribution-NonCommercial-NoDerivatives 4.0 International license (https://creativecommons.org/licenses/by-nc-nd/4.0/).

There are infrequent reports on right ventricular hydatid cysts in the literature, and this example contributes to the limited amount of rare but important presentations in the literature. For example, Aljaber et al. reported on a 23-year-old female who had two right ventricular hydatid cysts with firm attachments to the interventricular septum, requiring surgical excision (Figures 4-5) [15]. Lyazidi et al. also reported a case of a right ventricular, free wall hydatid cyst in a 14-year-old female with exertional dyspnea that was treated with a successful surgical approach [16]. These cases offered potential diagnostic and therapeutic challenges with common clinical features, nonspecific symptoms, and the use of a multimodal imaging approach to guide surgical planning. Intrathoracic extension to more than one compartment-lungs, pleura, mediastinum, and pericardium-is exceedingly rare and implies either primary hematogenous dissemination or secondary transdiaphragmatic spread from hepatic cysts [15]. Vesicular, grape-like congeries in sputum, or hydatidoptysis, are a pathognomonic but infrequent sign, most often the result of rupture of a pulmonary cyst into the bronchial tree [16].

*Echinococcus* serology remains a helpful diagnostic adjunct, and enzyme-linked immunosorbent assays (ELISA) and indirect hemagglutination tests are satisfactory in terms of sensitivity, especially when interpreted in combination with imaging [17]. Transthoracic echocardiography here provided early suggestion of cardiac involvement, and contrast-enhanced CT delineated the extent of thoracic disease, allowing prompt surgical referral. 

Treatment of disseminated thoracic hydatidosis is multidisciplinary. The primary treatment of cardiac and pericardial cysts, especially in symptomatic or complicated patients, is surgery [8]. Albendazole is used adjunctively pre- and post-operatively to reduce recurrence and treat inoperable lesions [18].

This case contributes to the limited literature on diffuse thoracic echinococcosis with right ventricular involvement. Because of the potentially fatal complications of cardiac hydatid disease-embolization, arrhythmias, or rupture-early recognition based on a combination of clinical suspicion, serology, and multimodal imaging is imperative [19].

## Conclusions

This is an uncommon presentation in an old patient of disseminated thoracic hydatidosis with intracardiac extension. The rare site in the right ventricle combined with pleuropulmonary, mediastinal, and pericardial extensions makes the diagnosis very difficult and calls for advanced imaging. Early diagnosis and a multidisciplinary approach reduce morbidity and prevent life-threatening complications, especially in endemic areas where this parasitic malady is ignored. The clinician should be aware that the early application of serological testing and echocardiography in patients presenting with unexplained thoracic symptoms, especially in endemic regions, can drastically improve outcomes through earlier surgical referral prior to complications such as embolization or cyst rupture. Different presentations of the disease in distinct geographical regions, like not involving the liver, should also modify the clinician's clinical suspicion as well as their diagnostic workup even without typical risk factors. This case reinforces the need for high clinical vigilance and recognition to utilize imaging and serology for atypical presentations of respiratory or cardiac phenomena.
